# Supplementary material for: Co-evolution within structured bacterial communities results in multiple expansion of CRISPR loci and enhanced immunity
Source: eLife. 2020 Mar 30;9:e53078. doi: 10.7554/eLife.53078 (PMC7105378; doi:10.7554/eLife.53078)
Supplement: Supplementary file 1. [file elife-53078-supp1.docx]

| Primer name | Sequence (5’-3’) |
| --- | --- |
| H54 | AAAACAGAGCGCAATTAATTATTGCGGATATTCCT |
| H237 | GGCGTACTGATGAAGATTATTTCTTAATAACTAAAAATATGG |
| NP389 | GCAAGAAGAAATCAACCAGCGC |
| NP390 | GAACCACGTGAACTATATGATTTTCCG |
| H463 | AAAACAAATACTGCTAACAAAATTAGTGATTGTAC |
| H493 | AAAACATTGCAGCTGTTAGCAGTGTGTTAAGTAAT |
| H40 | AAAACATCAAAGAATACTTTGCTGGTGTCGACTGGG |
| NP255 | AAAACCTTTTTCTTCAATTGGTCGACGTTTGAATAT |
| NP170 | CGATCAGGAATTGAGACACCTCAATATATACTTGCTGGTG |
| AV363 | TATCCAATTTTCGTTTGCCTCTTGCTTAAACCTATCC |
| NP331 | GAATAGTTACGATGATATCCTGCGCC |
| H462 | AAACGTACAATCACTAATTTTGTTAGCAGTATTTG |
| NP180 | CGATCCTTTAAATGTTTTAAAAGAATAGCATCATTTGGGG |
| H485 | AAAACTCTTGCTTTAATAAAATCTTTTGTTTTAGC |
| W1051 | GAACCTACGTCCGTAATGCTAGGATTTGCAAATTTCTTA |
| NP182 | CGATCAAACAGTGACAGAAACTATTGAGTACGAGGAGGTA |
| NP279 | AAAACTTTAACTAATAATAACAATACTAATAATGA |
| W1087 | GAACCACCCATATCATCTAGTACAAGTAAATCAATATCA |
| H501 | GACTGTTTCTCTCATTGTTGCG |
| H471 | AAAACAGAGTATTATCAACGACCTGAAGTTAAAAA |
| H122 | AAAACATATGGTGGGTAGTTTAATTCTTGCATTTT |
| H135 | AAAACAATTCCTTCTTCGTCTGCTATCTTTCGCAC |
| AV462 | GGAGCACGCACACCAATTTAAG |
| H453 | AAAACTTGTTAAATCATATGCGTCAATTAAGTGAA |
| H477 | AAAACTTTTAGCAATATGATTCTAGGCCATAACGG |
| NP313 | AAACAAAGTTTCTAACTAAAAATTGATGTGCAGACG |
| NP265 | AAAACACTTATGCCGTTTCTATACTTCACTACAGCA |
| H613 | CCTGTTCGTCTGTGTTCTCTTCAATCCATTCAT |

**Supplementary table. Oligonucleotide primers used in this study.**
